# Theoretical Calculations of Refractive Properties for Hg_3_Te_2_Cl_2_ Crystals

**DOI:** 10.1186/s11671-016-1476-8

**Published:** 2016-05-16

**Authors:** O. V. Bokotey

**Affiliations:** Faculty of Physics, Uzhhorod National University, Pidhirna Str., 46, Uzhhorod, 88000 Ukraine

**Keywords:** Corderoite structure, Wyckoff positions, Polarizability, Refractive index

## Abstract

This paper reviews the optical properties, such as refractive index, optical dielectric constant, and reflection coefficient of the Hg_3_Te_2_Cl_2_ crystals. The applications of the Hg_3_X_2_Y_2_ crystals as electronic, optical, and optoelectronic devices are very much determined by the nature and magnitude of these fundamental material properties. The origin of chemical bonding in the crystals is very important for definition of the physical and chemical properties. The main structural feature of the Hg_3_X_2_Y_2_ crystals is the presence of covalent pyramids [XHg_3_] and linear X-Hg-X groups. Optical properties are calculated according to the model proposed by Harrison. The refractive index in the spectral region far from the absorption edge is determined within the generalized single-oscillator model. The calculated results are found to be in good agreement with experimental data.

## Background

The crystal structure of mercury chalcogenhalogenides possesses a lot of features which promote the appearance of structural instabilities. All chalcogenchlorides and Hg_3_Te_2_Br_2_ are crystallized in the corderoite structure and described by the T^5^-I2_1_3 space symmetry group [1–3]. The main feature of synthesized and natural mercury chalcogenhalogenides is the formation of numerous polymorphic modifications and existence of isomorphic substitutions in chalcogen and halogen anion sublattices. Interest in them is caused by the ability to form continuous raw of solid solutions that allows them for different variations of physical and chemical properties. Hg_3_X_2_Y_2_ crystals are characterized by physical properties such as optical activity, high refractive index, transparency in visible and IR-range, photoconductivity, and electrooptical effect. Hg_3_X_2_Y_2_ (X = S, Se, Te; Y = F, Cl, Br, I) compounds, their synthesis and polymorphism are investigated in Refs. [1–10]. Optical activity and refractive index are studied in Refs. [1, 10–14]. Raman and IR spectra are investigated in Refs. [2, 15, 16]. The detailed research results of absorption edge are presented in Refs. [17–19]. These structural and optical properties of mercury chalcogenhalogenides crystals can be combined in modelling of physical and chemical characteristics of nanomaterials for nonlinear optic devices.

The present paper is aimed at the theoretical studies of the refractive index and reflection coefficient in Hg_3_Te_2_Cl_2_ crystals. It should be noted that the refractive index is one of the fundamental properties of a material because it is closely related to the electronic polarizability of ions and the local field inside the material. Besides that, theoretical study of refractive index gives more detailed information about crystals properties. The refractive index evaluation is of considerable importance for applications in integrated optic devices, where materials refractive index is the key parameter for device design. The calculated results are compared with available experimental data. Such results are obtained for the first time.

### Structure of Hg_3_X_2_Y_2_ Crystals

A special feature of all modifications of Hg_3_X_2_Y_2_ (X = S, Se, Te; Y = F, Cl, Br, I) compounds is a stronger ordering of the anions, as compared to the cations, owing to the strong covalent Hg–X bonds, which form various configurations with virtually the same «fixed» bond-lengths. The studied structures consist of strong covalent and connected pyramids [XHg_3_] and linear X–Hg–X groups which in different structural types form different spatial connections. At the same time, the presence of the «hinge-joint» bonds in the covalent –X–Hg–X–Hg–X– radical results in the appearance of many polymorphic modifications [7]. The structure of the α-Hg_3_S_2_Cl_2_ type is realized in the cases, when the chalcogen anion size is more than halogen anion size S^2−^ (0.182 nm), Se^2−^ (0.193 nm), Te^2−^ (0.211 nm) >Cl^−^ (0.181 nm); Te^2−^ (0.211 nm) >Br^−^ (0.196 nm) [20].

**Fig. 1:**
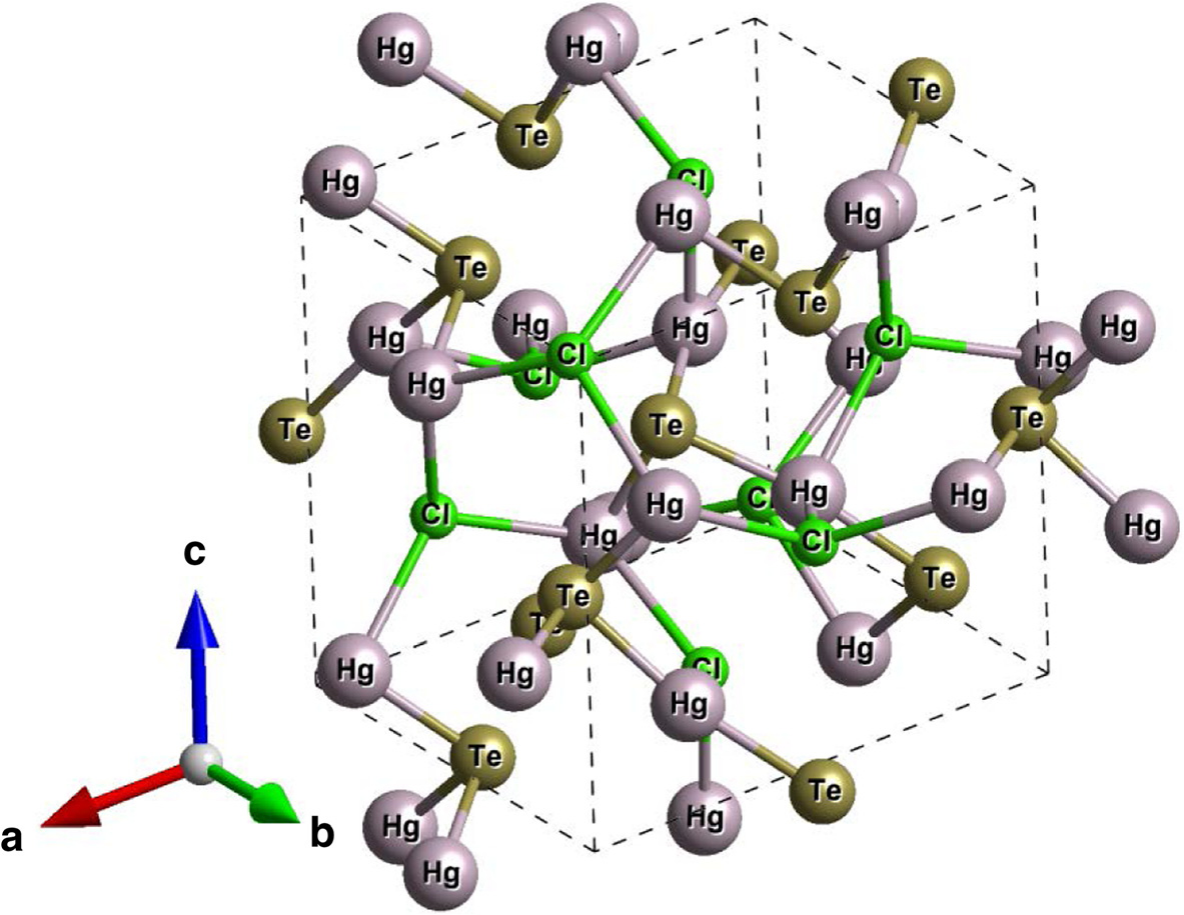
Crystal structure of Hg_3_Te_2_Cl_2_

Hg_3_X_2_Y_2_ gyrotropic crystals are characterized by the structure with spiral chains. The atoms are located on the upward or downward double spirals in the structure (Fig. 1). There are some groups of atoms in the structure of Hg_3_X_2_Y_2_ crystals, which act as an optically active chromophore. [HgX_2_Y_4_] octahedra form spiral chains with triple spiral axis in the (111) direction of elementary cube. A characteristic feature of all compounds is the presence of two sets of octahedral spirals with different radii and twisting directions. They are located side by side, oriented in the same direction, and consistently alternated. The spiral which is twisted counterclockwise has a larger radius. The step of both spirals is equal. Sign of Hg_3_Te_2_Cl_2_ crystal rotation is caused by the strong influence of Х^VI^ and Y^VII^ atoms on the symmetry of local crystal field and the polarization medium as a result. Angle Y-Hg-Y plays a decisive role because averaging over the four X-Hg-Y angles results to the same value in all compounds. Chalcogen atom replacement leads to a significant variation of Y-Hg-Y angle and stronger displayed on the optical activity value than replacement of halogen atom [1]. The unique structural features of Hg_3_X_2_Y_2_ crystals are conditioned by fundamental properties of conformational polymorphism.

Hg_3_Te_2_Cl_2_ crystals belong to the ternary chalcogenhalogenide compounds. The number of formula units in the unit cell is Z = 4. The unit cell of Hg_3_Te_2_Cl_2_ contains 14 atoms, including six Hg^II^, four Te^VI^, and four Cl^VII^. Other crystal parameters are presented in Table 1. Hg_3_Te_2_Cl_2_ structure is characterized by the following set of possible nuclear positions: С_2_(4), С_2_(6), С_1_(12). Atoms occupy the positions: 12Hg (b) [x 0 0.25] (x = 0.31), 8Te (a) [x x x] (x = 0.28), 8Cl (a) [x x x] (x = 0.025). The lattice parameter is a = 9.33 Å [2, 15]. The Wyckoff positions define by the next set:

$$ \begin{array}{l}12\mathrm{B}(2)\hbox{--} \left\{\mathrm{x},\kern0.5em 0,\kern0.5em 1/4\right\},\kern0.5em \left\{1/2-\mathrm{x},\kern0.5em 0,\kern0.5em 3/4\right\},\kern0.5em \left\{1/4,\kern0.5em \mathrm{x},\kern0.5em 0\right\},\kern0.5em \left\{0,\kern0.5em 1/4,\kern0.5em \mathrm{x}\right\},\kern0.5em \left\{3/4,\kern0.5em 1/2-\mathrm{x},\kern0.5em 0\right\},\kern0.5em \left\{0,\kern0.5em 3/4,\kern0.5em 1/2-\mathrm{x}\right\};\hfill \\ {}8\mathrm{A}(3)\hbox{--} \left\{\mathrm{x},\kern0.5em \mathrm{x},\kern0.5em \mathrm{x}\right\},\kern0.5em \left\{1/2+\mathrm{x},\kern0.5em 1/2-\mathrm{x},\kern0.5em -\mathrm{x}\right\},\kern0.5em \left\{1/2-\mathrm{x},\kern0.5em -\mathrm{x},\kern0.5em 1/2+\mathrm{x}\right\},\kern0.5em \left\{-\mathrm{x},\kern0.5em 1/2+\mathrm{x},\kern0.5em 1/2-\mathrm{x}\right\}.\hfill \end{array} $$

**Table 1:** Crystal data of Hg_3_Te_2_Cl_2_

Chemical formula	Hg_3_Te_2_Cl_2_
Space group	I2_1_3
Formula units per cell	4
a (nm)	0.9326
b (nm)	0.9326
c (nm)	0.9326
*Z*	4
Ratio of anions radii *X* ^2−^/Hal^−^	1.17
Structural type	*α*-Hg_3_S_2_Cl_2_

There are two types of chemical bonds in investigated crystals: the covalent—between mercury and chalcogen atoms and ionic—between mercury and halogen atoms [18–20]. The Hg–Cl bond length is 2.99 Å, while the Hg–Te bond length is 2.65 Å. Besides that, many interesting chemical bond aspects connected with existence of intrinsic defects. As Hg–Te is the main chemical bond, such structure interpretation reflects physical and chemical properties of the Hg_3_Te_2_Cl_2_ crystals.

## Methods

For calculation of the refractive index, the Harrison bonding-orbital method was used [20, 21]. Using the Harrison bonding-orbital theory is irreplaceable for calculation of parameters, which describe the structure of energy bands, as well as for understanding the physical nature of this structure. The essence of bonding-orbital method consists in the description of the localized charges on the basis of interactions between electronic orbitals of structure atoms. As follows from the theory, analysis of covalent and ionic crystals is almost always based on the description of electron conditions in crystal in the form of linear combination of electronic orbitals. The complex approach to the investigation of optical properties includes some steps: neglect all matrix elements between bonding and anti-bonding states; reduce Hamiltonian matrix to two matrixes, one of which is constructed on valence band conditions, and another—on conductive band conditions; use Wannier functions for zeroing of matrix elements between bonding and anti-bonding states in matrix. As the result, one can obtain the diagonal matrix elements, which correspond to Wannier energy levels, as well as matrix elements between the bonding states, responsible for splitting of these levels in bands.

Most optical properties of semiconductors are integrally related to the particular nature of their electronic band structures. Band structure is in turn related to the type of crystallographic structure, the particular atoms, and their bonding. Both the valence band and conduction band states are important for prediction of the refractive index or optical dielectric constant.

## Results and Discussion

The crystal optical investigations provide an important information concerning the nature and the properties of Hg_3_X_2_Y_2_ crystals. The refractive index is a very important physical parameter related to the microscopic atomic interactions. From the theoretical stand point, there are basically two different approaches in viewing this subject: on one hand, considering the crystal as a collection of an electric field, the refractive index will be related to the density and the local polarizability of these entities. On the other hand, considering the crystalline structure represented by a delocalized picture, the refractive index will be closely related to the energy band structure of the material, thorough quantum-mechanical analysis required is complicated and the results obtained are very particular [22–24]. The refractive index in the spectral region far from the absorption edge was determined within the generalized single-oscillator model. It makes possible to find the energies of filled electronic states using the Hartree–Fock values [21] for the valence levels in complex crystals. The approach becomes particularly useful when it is simplified by including only nearest-neighbor couplings and using universal parameters, which allows direct prediction of all properties [25].

According to the special points method [20, 21, 25, 26], any average of any property over the electronic energy band is replaced by the values at a single special wave number, the Baldereschi point *k*. That wave number is chosen such that the first few sets of Fourier components of the band vanish, just as one would choose a point halfway to the Brillouin zone boundary in a one-dimensional crystal. As follows from the theory, the interaction between orbitals of cation and anion is described by the energy:

1 $$ {E}_k=\frac{E_s+{E}_p}{2}\pm \sqrt{{\left(\frac{E_s-{E}_p}{2}\right)}^2+f{(k)}^2{{\mathrm{V}}_{\mathrm{sp}\upsigma}}^2}, $$

where *f* (*k*) depending upon the relative phase and orientation of neighboring orbitals.

The first and second terms under the root sign are energies *V*_3_ and *V*_2_. The bonding-orbital method based on definition of bond energy. Covalent bond energy *V*_2_ [25] calculates as:

2 $$ {V}_2 = \sqrt{f(k)}{V}_{\mathrm{b}}, $$

For different interaction cases *V*_b_ equals:

3 $$ \begin{array}{ccc}\hfill {\mathrm{V}}_{\mathrm{sp}\upsigma}=1.42\left(\frac{{\mathrm{\hbar}}^2}{{\mathrm{md}}^2}\right),\hfill & \hfill {\mathrm{V}}_{\mathrm{pp}\upsigma}=2.22\left(\frac{{\mathrm{\hbar}}^2}{{\mathrm{md}}^2}\right),\hfill & \hfill {\mathrm{V}}_{\mathrm{pp}\uppi}=-0.63\left(\frac{{\mathrm{\hbar}}^2}{{\mathrm{md}}^2}\right)\hfill \end{array}, $$

The dominant bands are based upon p-states and s-states, and contributions to the properties depend upon the coupling between neighboring orbitals and their energy difference ${E}_s-{E}_p$ taken from Hartree–Fock term values [20]. According to [25], the ionic bond energy *V*_3_ equals to half the term-value difference:

4 $$ {V}_3=\left({E}_s-{E}_p\right)/2, $$

By analysis, the polarizability and susceptibility χ, it is possible to calculate the refractive index. The treatment of bond polarizabilities use cluster approximation based upon special point. It is necessary to calculate the coupling of each occupied band state to every empty state, evaluate the change in charge on every atom, and sum the contributions of the resulting perturbed electron density to obtain a dipole and the exact polarizability. The cluster consists of different states on each of the two neighbors. The individual bond polarizability [25] can be calculated as:

5 $$ \alpha =\gamma \frac{\left(1-{\alpha}_p\right){\alpha}_p{e}^2{d}^2}{\left(1+{\alpha}_p\right){V}_3}, $$

where ${\alpha}_p$ is polarity and defines as:

6 $$ {\alpha}_p=\frac{V_3}{\sqrt{V_2^2+{V}_3^2}}, $$

The optical dielectric susceptibility is calculated as the sum of polarizabilities of each bond type by their number per unit volume. Knowledge of susceptibility allows to predict the refractive index. The optical dielectric constant and refractive index are very important in determining the electric and optical properties of the materials. The imaginary part of the dielectric constant shows how a dielectric absorbs energy from an electric field caused by dipole motion. The real part of the dielectric function can be derived from the imaginary part by the Kramers–Kronig relationship. The refractive index *n* (*ω*) is given by Eq. (7):

7 $$ n\left(\omega \right) = {\left[\frac{\varepsilon_1\left(\omega \right)}{2}+\frac{\sqrt{\varepsilon_1^2\left(\omega \right)+{\varepsilon}_2^2\left(\omega \right)}}{2}\right]}^{\raisebox{1ex}{$1$}\!\left/ \!\raisebox{-1ex}{$2$}\right.} $$

where ${\varepsilon}_1$ and ${\varepsilon}_2$ are the real and imaginary components of the dielectric function, respectively. At low frequency *ω* = 0, the dielectric constant of a material is related to the refractive index by Eq. (8):

8 $$ n = \sqrt{\varepsilon }, $$

Reflection coefficient can be calculated as:

9 $$ R = \frac{{\left(n-1\right)}^2}{{\left(n+1\right)}^2} $$

Thus, using Harrison’s model, the refractive index and reflection coefficient are calculated. The results are presented in Table 2. The following notations are used in Table 2: *d* is the bond length, *Z* is the coordination number, *V*_2_ is the energy of covalent bond, *V*_3_ is the energy of ionic bond, *γ* is the coefficient, *α* is the polarizability, *χ* is the susceptibility, *n* is the refractive index, and *R* is the reflection coefficient.

**Table 2:** Calculated parameters of Hg_3_Te_2_Cl_2_ crystals

Bond type	*d*, Å	*N*_*c*_	*V*_2_, *eV*	*V*_3_, *eV*	*γ*	*α*, Å^3^	*Χ*	*n*	*R*
Hg–Te	2.65	4	3.08	2.42	1.5	25.3	0.25	2.3	0.16
Hg–Cl	2.99	4	2.42	3.34	1.5	7.59	0.08		

It should be noted that theoretical calculations using complex approach always give underestimated values in comparison with experimental data. Refractive index varies from 3.06 at 4650 Å to the vicinity of the absorption edge, then starts to level off and reaches 2.68 at 7000 Å [4] for Hg_3_Te_2_Cl_2_ crystals. The calculated value of the refractive index is *n*_theor_ = 2.3. A good agreement is observed with experiment [4]. This is verified by the calculation of the optical dielectric constant which depends on the refractive index.

## Conclusions

The refractive index calculations of the Hg_3_Te_2_Cl_2_ crystals using the Harrison bonding-orbital method are presented. Satisfactory agreement between experimental data and calculation results is obtained. It is shown that this approximation allows to analyze the optical properties of the Hg_3_X_2_Y_2_ (X = S, Se, Te; Y = F, Cl, Br, I) crystals. It is evident that structural features, covalent Hg–X chemical bonds of the Hg_3_X_2_Y_2_ crystals reflect their physical and chemical properties. Optical properties play a vital role in understanding the structure and the nature of chemical bonding in the crystals. Refractive index is very important magnitude that decided the optical and electronic behavior of crystals used for possible applications. It can be concluded from the present and previous studies that Hg_3_Te_2_Cl_2_ crystals are perspective nanomaterials for application in nonlinear optical devices. Finally, it should be noted that results obtained in this paper confirm the possibility of their application for analysis of the optical properties of complex compounds.
